# The Complex Biology of EIF3E (int6): From Its Original Discovery to *EIF3E::RSPO2* Fusions in Solid Tumors and Lymphoma Susceptibility

**DOI:** 10.3390/cancers18193120

**Published:** 2026-09-26

**Authors:** Antonio Marchetti, Fiamma Buttitta

**Affiliations:** Department of Medical, Oral and Biotechnological Sciences, “G. d’Annunzio” University of Chieti-Pescara, 66100 Chieti, Italy; fbuttitta@unich.it

**Keywords:** EIF3E, int6, *EIF3E::RSPO2* fusion, selective translation, proteostasis, lymphoma susceptibility, cancer, DNA repair

## Abstract

Over the past three decades, the biology of EIF3E (int6) has emerged as far more complex than originally anticipated. Initially identified as a common integration site of Mouse Mammary Tumor Virus (MMTV) and subsequently recognized as a component of the eukaryotic translation initiation factor 3 (eIF3) complex, EIF3E is now understood as a multifunctional regulator involved in selective mRNA translation, proteostasis, genome stability, cellular stress responses, and tissue homeostasis. Recent studies have further extended this framework by demonstrating that conditional *Eif3e* deficiency promotes lymphoma susceptibility in mice, providing in vivo evidence linking EIF3E-dependent translational regulation to immune homeostasis and cancer development. This focused review retraces the scientific journey of EIF3E from its discovery as int6 to the progressive definition of its diverse biological functions and context-dependent roles in cancer. Particular attention is given to the historical relationship between int6 (EIF3E) and int7 (RSPO2), whose unexpected reunion in recurrent *EIF3E::RSPO2* fusions in human solid tumors provides a unique bridge between classical retroviral insertional mutagenesis and contemporary cancer genomics. While transcriptional activation of RSPO2 through EIF3E regulatory elements represents the established oncogenic mechanism of the fusion, whether disruption of one EIF3E allele exerts additional biological effects remains unknown. Together, these observations highlight EIF3E as a nexus between selective translation, cellular homeostasis, and cancer, while emerging links between epigenomic, epitranscriptomic, and translational regulation open new perspectives for understanding its context-dependent functions in tumorigenesis.

## Simple Summary

EIF3E is a protein originally discovered through studies of mouse mammary tumors and was subsequently identified as a component of the cellular machinery controlling protein synthesis. Research has since revealed broader functions for EIF3E in the selective production and quality control of proteins, cellular homeostasis, and cancer-related processes. Recent findings have further linked loss of EIF3E function to lymphoma susceptibility in mice. Remarkably, EIF3E has also re-emerged in human cancer through a recurrent fusion with RSPO2, another gene discovered in the same experimental tumor model. In the *EIF3E::RSPO2* fusion, the established oncogenic effect is driven by RSPO2 overexpression under EIF3E regulatory control; whether disruption of one EIF3E allele exerts additional biological effects remains unknown. This review retraces the scientific history of EIF3E and integrates these apparently distinct discoveries. Understanding these connections may provide new perspectives on tumor development and the biological consequences of EIF3E alterations.

## 1. Introduction

### 1.1. MMTV Insertional Mutagenesis and the Identification of int6

The eukaryotic translation initiation factor 3 subunit E (EIF3E), initially identified as the Mouse Mammary Tumor Virus (MMTV) common integration site int6, was first recognized during studies of retrovirus-induced mammary tumorigenesis. MMTV represents one of the best-characterized models of retrovirus-induced carcinogenesis. Unlike acutely transforming retroviruses carrying viral oncogenes, MMTV promotes mammary tumor development predominantly through insertional mutagenesis, whereby integration of the proviral genome into host chromosomal DNA alters the expression of adjacent cellular genes. The pioneering studies of Nusse and Varmus established that recurrent proviral integration could activate specific host genes, providing the first demonstration that retroviral insertion represented a powerful strategy for identifying cancer-associated genes rather than merely a mechanism of viral replication [1,2].

As additional MMTV-induced mammary tumors were molecularly characterized, it became evident that proviral integrations were not randomly distributed throughout the genome but clustered within restricted chromosomal regions in independent tumors. This observation led to the concept of common integration sites (CISs), genomic loci repeatedly selected during tumor evolution because alteration of nearby genes conferred a biological advantage to the transformed cell. During the following years, systematic analysis of these recurrent integration sites led to the identification of several genes that proved to play fundamental roles in development and cancer, including int1, subsequently recognized as Wnt1, int2 (Fgf3) and int3 (Notch4) [1,3,4]. As emphasized in the historical reviews by Callahan and colleagues, the MMTV model thus evolved into one of the most productive experimental systems for cancer gene discovery, anticipating the identification of numerous signaling pathways later recognized as central regulators of tumorigenesis [5,6].

Within this experimental framework, Marchetti et al. identified a previously unknown common integration site, designated int6, during the analysis of MMTV-induced mammary preneoplastic lesions and tumors [7]. Unlike the previously characterized *int* loci, int6 did not encode an obvious growth factor or signaling molecule. Instead, molecular cloning demonstrated that it corresponded to a highly conserved and ubiquitously expressed gene, whose transcript was disrupted by proviral insertion, generating a truncated messenger RNA predicted to encode a C-terminally deleted protein. These observations immediately distinguished int6 from the other MMTV integration sites and suggested that retroviral insertion was targeting a gene with a previously unrecognized cellular function rather than a conventional signaling regulator.

Although the biological significance of int6 remained uncertain at the time of its discovery, the identification of a highly conserved and widely expressed gene as a recurrent MMTV integration site raised an important question: why would disruption of a gene apparently involved in fundamental cellular processes contribute to mammary tumorigenesis? Addressing this question would require identification of the molecular function of the INT6 protein, an objective that was achieved only a few years later with its recognition as a component of the eukaryotic translation initiation factor 3 (eIF3) complex.

### 1.2. From int6 to EIF3E: A Translation Initiation Factor

A major breakthrough came only a few years later, when biochemical studies of eukaryotic translation initiation factor 3 converged unexpectedly with the field of MMTV insertional mutagenesis. While characterizing the subunit composition of purified human eIF3, Asano and colleagues cloned the cDNA encoding its 48-kDa subunit and found that it corresponded to the previously identified int6 gene. The recombinant protein comigrated with the native p48 component of purified eIF3 and was associated with other subunits of the complex, providing direct evidence that INT6 was a bona fide component of the translation initiation machinery [8].

This finding connected two previously independent lines of investigation. A gene discovered through retroviral insertional mutagenesis was now recognized as encoding a component of eIF3, one of the largest mammalian translation initiation factors. Contemporary biochemical studies described eIF3 as a multisubunit complex that binds the 40S ribosomal subunit early during initiation and promotes the recruitment of initiator methionyl-tRNA and mRNA. Comparative analyses also showed both conservation and substantial divergence in eIF3 composition across eukaryotes, placing INT6 within a broader evolutionary framework rather than defining it as an isolated mammalian protein [9].

The molecular characterization of human eIF3 subunits further clarified the organization of the complex. Cloning and sequence analysis of several components identified conserved structural motifs potentially involved in RNA binding and macromolecular assembly, while confirming the heterogeneous nature of mammalian eIF3. Within this emerging architecture, the former INT6 protein was designated p48 according to its apparent molecular mass, a terminology that coexisted for several years with the name derived from its discovery as an MMTV integration site [8].

The coexistence of molecular-mass designations, gene names and organism-specific terminology eventually made comparisons among eIF3 complexes increasingly difficult. In 2001, Browning and colleagues proposed a unified nomenclature in which the subunits were assigned consecutive letters, broadly following their apparent molecular masses in the mammalian complex. Under this system, p48/INT6 became eIF3e, encoded by the *EIF3E* gene. The new terminology formally joined the two identities of the molecule: int6, the gene discovered through viral oncogenesis, and *EIF3E*, the gene encoding a defined subunit of the eIF3 complex [10].

Initially, this reassignment appeared to place EIF3E within the conventional framework of general translation initiation. Yet the identification of INT6 as an eIF3 subunit did not resolve the biological questions raised by its recurrent disruption in MMTV-induced tumors. Rather, it reformulated them. The central issue was no longer simply why MMTV targeted int6, but whether alteration of a specific eIF3 component could affect cellular functions in ways not explained by a uniform reduction in protein synthesis. That question would guide the subsequent investigation of EIF3E and gradually reveal a biological profile extending beyond its initial classification as a constitutive component of the translation apparatus, as summarized in Figure 1.

### 1.3. Beyond a General Translation Initiation Factor

As a component of the eukaryotic translation initiation factor 3 (eIF3), EIF3E was naturally assumed to participate in the general process of protein synthesis, and its involvement in MMTV-induced mammary tumorigenesis was interpreted within this framework [8,16].

The first evidence challenging this interpretation emerged from genetic studies in model organisms. Morris-Desbois and colleagues reported that studies in *Schizosaccharomyces pombe* had revealed selective phenotypes associated with Int6 dysfunction, including slow growth under specific culture conditions, altered responses to environmental and pharmacological stress, and defects in chromosome segregation [17]. Rather than reflecting the generalized cellular impairment expected from disruption of an essential translation initiation factor, these observations suggested that the biological functions of Int6 extended beyond maintaining global protein synthesis.

These observations were subsequently reinforced by studies in *S. pombe*. Yen and colleagues demonstrated that Int6 plays a selective role in cell division and mitotic fidelity, further supporting the view that its biological functions could not be explained solely by its participation in basal translation initiation [18].

At approximately the same time, biochemical studies began to reveal unexpected molecular interactions involving EIF3E, suggesting that its cellular functions might extend beyond its established role within the eIF3 complex [19,20]. Together with the selective phenotypes observed in experimental models, these findings progressively weakened the view of EIF3E as merely a constitutive component of the translation initiation machinery and supported the emerging concept of a protein involved in specific regulatory programs [17,18,19,20].

This conceptual shift represented a pivotal moment in EIF3E research. The central question was no longer whether EIF3E functioned as an eIF3 subunit—this had already been firmly established—but how a component of the translation machinery could influence such diverse cellular processes. Addressing this question led directly to the investigation of the molecular relationships between EIF3E, translation and protein homeostasis.

### 1.4. EIF3E at the Interface Between Translation and Protein Homeostasis

Early biochemical studies provided the first molecular basis for this broader interpretation of EIF3E function. An important milestone came from studies in plants, where homologues of EIF3E were found to associate with the COP9 signalosome (CSN), a multiprotein complex structurally related to eIF3 and critically involved in ubiquitin-mediated regulatory pathways. Shortly thereafter, Yahalom and colleagues demonstrated that Arabidopsis eIF3e interacts both with eIF3c and with the CSN7 subunit of the COP9 signalosome, suggesting that EIF3E could participate in distinct molecular assemblies rather than functioning exclusively as a constitutive component of eIF3 [20,21,22].

Parallel investigations in yeast further refined this view. Studies in *Schizosaccharomyces pombe* confirmed that Int6 was an authentic component of eIF3 but also demonstrated that its deletion did not abolish global translation initiation. Instead, loss of Int6 produced selective defects in cell physiology. Additional work showed that Int6 participates in the proper organization and stability of the eIF3 complex, reinforcing the concept that individual eIF3 subunits possess distinct biological properties despite belonging to the same macromolecular assembly [16,19,23].

A major conceptual advance followed with the demonstration that mammalian INT6 physically associates not only with eIF3 but also with the COP9 signalosome and the 26S proteasome. Hoareau Alves and colleagues proposed that INT6 occupies a unique position among the three principal PCI (Proteasome–COP9–Eucaryotic Initiation factor 3) complexes, which share conserved structural motifs despite performing different cellular functions. This observation suggested, for the first time, that EIF3E might coordinate molecular events occurring at the interface between protein synthesis and protein degradation [20].

The biological significance of these interactions was subsequently established by functional studies demonstrating that Int6 regulates proteasome integrity and activity. Yen and colleagues showed that loss of Int6 disrupts proteasome assembly through altered localization of the proteasome subunit Rpn5, resulting in the accumulation of polyubiquitinated proteins and mitotic regulators. These findings transformed the interpretation of the earlier biochemical observations by demonstrating that the association between EIF3E and the proteasome reflects a functional regulatory mechanism rather than a simple physical interaction [18,24].

During the following years, evidence accumulated that EIF3E contributes to the selective control of translation itself. EIF3E was shown to participate in distinct eIF3 assemblies displaying differential association with subsets of mRNAs, providing one of the earliest indications that the eIF3 complex could exert transcript-selective regulatory functions [25]. These observations suggested that EIF3E might contribute to determining the specificity of translational regulation.

Subsequent studies integrated these apparently independent observations into a coherent mechanistic framework. In human mammary epithelial cells, depletion of EIF3E simultaneously impaired proteasome assembly, altered ubiquitin homeostasis and selectively affected translation of specific transcripts, demonstrating that the protein regulates both protein synthesis and protein degradation within the same cellular context [26]. These findings provided compelling evidence that the dual involvement of EIF3E in translation and proteostasis represents an intrinsic biological property rather than the consequence of unrelated molecular interactions.

More recently, advances in ribosome profiling and structural biology have profoundly expanded this model. Contrary to the classical view that eIF3 dissociates immediately after translation initiation, Lin and colleagues demonstrated that eIF3, including EIF3E, remains associated with 80S ribosomes during early elongation on hundreds of transcripts encoding mitochondrial, membrane-associated and protein complex components. This prolonged association facilitates recruitment of molecular chaperones and quality-control factors, thereby promoting co-translational folding and organelle homeostasis [27]. Recent studies further demonstrated that retention of eIF3 on translating ribosomes is directed by specific 5′ untranslated region elements and is required for efficient co-translational folding of endoplasmic reticulum membrane proteins, providing a direct molecular mechanism linking EIF3E to protein quality control during translation [28].

Collectively, these studies have fundamentally reshaped the biological interpretation of EIF3E. Initially regarded as a structural subunit of the translation initiation machinery, EIF3E is now recognized as a multifunctional coordinator that integrates selective translation with protein folding, ubiquitin-dependent degradation and maintenance of cellular proteostasis. This conceptual evolution provides the mechanistic basis for understanding why alterations of EIF3E affect such a broad spectrum of cellular processes and pathological conditions [20,26,27,28].

### 1.5. From Protein Homeostasis to Cellular Homeostasis: Consequences of EIF3E Dysregulation

The progressive recognition that EIF3E coordinates selective translation, protein quality control and proteostasis naturally led to a broader question: how do alterations of EIF3E affect cellular behavior? Experimental evidence accumulated over the past two decades has shown that EIF3E dysregulation affects multiple interconnected cellular programs, including proliferation, differentiation, stress adaptation and tissue homeostasis [29,30].

Early genetic studies in model organisms showed that disruption of Int6 produced selective phenotypes rather than generalized translational failure. In *Schizosaccharomyces pombe*, deletion of *int6* caused only moderate inhibition of global protein synthesis while affecting responses to specific growth conditions and spore formation [23]. Together with subsequent evidence linking EIF3E to protein homeostasis and cellular responses to stress, these observations reinforced the emerging concept that EIF3E acts as a regulatory component controlling specific biological pathways [29,30].

As mechanistic understanding improved, the spectrum of cellular abnormalities associated with EIF3E dysfunction expanded considerably. Defective proteasome function and altered degradation of key regulatory molecules were associated with mitotic abnormalities and defects in chromosome segregation, establishing a connection between EIF3E deficiency, disruption of protein homeostasis and loss of normal cell-cycle control [20,29].

Subsequent investigations demonstrated that the consequences of EIF3E dysregulation extend to tissue organization. In mammary epithelial cells, depletion of EIF3E altered acinar morphogenesis, impaired epithelial organization and affected the translation of selected transcripts involved in ubiquitin metabolism and cellular architecture. These findings showed that perturbation of EIF3E-dependent regulation can translate molecular defects in protein synthesis and degradation into alterations of epithelial architecture [26].

Genome-wide analyses of translation further broadened this biological framework. EIF3E deficiency selectively compromises translation of mRNAs encoding mitochondrial proteins, membrane-associated proteins and components of large macromolecular complexes, with consequent impairment of mitochondrial function, oxidative metabolism and cellular adaptation to proteostatic stress [27]. More recent evidence linking eIF3 retention on translating ribosomes to co-translational protein folding further supports the concept that disruption of EIF3E-dependent translational programs can affect organelle homeostasis and cellular fitness [28].

Perhaps the most compelling demonstration that EIF3E governs integrated cellular programs rather than isolated molecular events has emerged from recent studies of immune homeostasis. Using conditional mouse models, recent work demonstrated that EIF3E establishes a translational checkpoint required for normal B-cell differentiation, immune homeostasis and protection against lymphoma development. Loss of EIF3E selectively reprograms translation of transcripts controlling lymphocyte activation, ultimately promoting lymphoid malignancy despite relatively modest effects on global protein synthesis. This work provides strong experimental evidence that selective translational regulation mediated by EIF3E can determine cell fate decisions in vivo and directly links disturbances in translational control to disease pathogenesis [15].

Collectively, these studies reveal a profound evolution in our understanding of EIF3E biology. What initially appeared to be a translation initiation factor has progressively emerged as a regulator of cellular homeostasis, integrating selective translation and protein quality control with mitochondrial function, stress adaptation and lineage-specific differentiation. This expanded biological framework provides the conceptual foundation for interpreting the diverse roles of EIF3E in human disease and, most notably, in cancer. The recent demonstration that EIF3E deficiency predisposes to lymphoma in vivo provides compelling physiological evidence that disruption of these integrated regulatory functions can drive disease development.

### 1.6. EIF3E and Cancer: An Evolving Interpretation

The identification of int6 as a recurrent site of mouse mammary tumor virus (MMTV) integration immediately raised the question of how disruption of this gene might contribute to mammary tumorigenesis. In the original lesions, the provirus was integrated within the gene in the opposite transcriptional orientation, generating truncated chimeric transcripts containing int6, intronic, and viral sequences. Because the second allele remained structurally intact, Marchetti et al. proposed that the viral insertion might either produce a dominant-negative alteration or otherwise modify the biological activity of INT6, thereby contributing to tumor development [7]. This hypothesis distinguished int6 from classical proto-oncogenes activated by retroviral insertion and established the first conceptual framework for investigating its role in cancer.

The first studies on the human homologue were designed to test this biological hypothesis. Loss of heterozygosity and reduced EIF3E expression were subsequently demonstrated in subsets of breast and non-small cell lung carcinomas, suggesting that impaired EIF3E activity may contribute to tumor development and supporting a tumor suppressor role for EIF3E [31,32,33].

The prognostic significance observed in early-stage lung cancer further strengthened the clinical relevance of these findings [32]. Together, these studies represented the first successful translation of observations from the MMTV model into human cancer.

Experimental studies performed during the following years further supported the involvement of EIF3E in neoplastic transformation. Truncated Int6 exhibited transforming activity in cultured mammary epithelial cells and fibroblasts [34,35], while transgenic mice expressing truncated Int6 in the mammary gland developed persistent epithelial hyperplasia and mammary tumors [36]. These findings demonstrated that structural alteration of Int6 is sufficient to promote mammary tumorigenesis in vivo and established a causal relationship between altered EIF3E function and malignant transformation in experimental systems.

As the biological functions of EIF3E became progressively clearer, however, a more complex picture emerged. Increased EIF3E expression was subsequently reported in breast carcinoma, colorectal cancer, progression of esophageal squamous cell carcinoma and glioblastoma, where EIF3E promoted proliferation, invasion or tumor cell survival through selective regulation of specific mRNA subsets [37,38,39,40].

Conversely, EIF3E depletion in non-transformed mammary epithelial cells induced epithelial-to-mesenchymal transition, disrupted acinar morphogenesis and impaired proteasome function, indicating that loss of EIF3E may also facilitate malignant transformation under specific biological conditions [33]. Additional studies demonstrated important roles for EIF3E in the DNA damage response, maintenance of genome integrity and regulation of cellular senescence [12,13]. More recent work further expanded these observations by showing that EIF3E contributes to selective translational programs involved in stemness, stress adaptation and therapeutic resistance in glioblastoma [41].

An additional link between EIF3E, proteostasis and tumor adaptation involves the hypoxia-inducible factor pathway. Int6/eIF3e was shown to interact selectively with HIF-2α and promote its proteasome-sensitive degradation through a mechanism independent of hypoxia and pVHL. Accordingly, Int6/eIF3e depletion stabilized HIF-2α and increased the expression of angiogenic mediators, including VEGF, angiopoietin and basic fibroblast growth factor [11]. Subsequent studies extended this axis to endothelial cells, where Int6/eIF3e silencing enhanced HIF-2α-dependent angiogenic and paracrine responses, including IL-6 and IL-8 signaling, suggesting that EIF3E perturbation may influence not only tumor-cell proteostasis but also the surrounding microenvironment [42]. The relationship is nevertheless context-dependent. In glioblastoma cells, EIF3E depletion reduced proliferation and survival and was accompanied by decreased HIF expression [40]. Together, these observations connect EIF3E-dependent proteostatic regulation with hypoxic and angiogenic adaptation while emphasizing that its effects on HIF signaling vary according to cellular context.

Collectively, these apparently divergent observations progressively shifted the interpretation of EIF3E beyond the traditional oncogene–tumor suppressor paradigm. Rather than functioning as a classical cancer gene, EIF3E emerged as a multifaceted regulator of selective mRNA translation and cellular homeostasis whose biological consequences depend on cellular context, differentiation state and the translational programs under its control. These apparently conflicting effects are likely shaped by cell type, differentiation and tumor stage, EIF3E dosage, and the nature of the alteration, including reduced or increased expression versus expression of truncated variants.

An unresolved mechanistic question is which transcript-specific features determine dependence on EIF3E. Defining these determinants will be important for understanding how changes in EIF3E abundance or function selectively remodel translational output in different cellular and tumor contexts.

This conceptual framework has been further reinforced by recent in vivo evidence. Conditional deletion of *Eif3e* in germinal-center B cells disrupts a translational checkpoint required for immune tolerance, resulting in immune dysregulation and the development of lymphoid malignancies [15]. Importantly, this phenotype arises through mechanisms distinct from those proposed in the original MMTV studies and from the transforming activity of truncated Int6, indicating that impairment of physiological EIF3E function may contribute to tumorigenesis through multiple biological pathways. Although obtained in a murine model, these findings considerably extend the biological framework established by earlier experimental studies, demonstrating that the physiological functions of EIF3E are required not only for translational and proteostatic homeostasis but also for maintaining immune homeostasis and protecting against malignant transformation.

This biological framework also provides the appropriate context for interpreting the relatively limited spectrum of recurrent EIF3E genomic alterations identified in human cancer and, most notably, the emergence of the *EIF3E::RSPO2* fusion as a recurrent event in human solid tumors.

## 2. *EIF3E::RSPO2* Fusion: Reconnecting int6 and int7 in Human Cancer

Although early studies demonstrated loss of heterozygosity and reduced *EIF3E* expression in subsets of human breast and lung carcinomas, suggesting that impaired *EIF3E* activity may contribute to tumor development [31,32,43], subsequent large-scale cancer genomic studies have shown that recurrent *EIF3E* mutations are rare, with no evidence of mutational hotspots or a characteristic pan-cancer mutational spectrum. Rather than being characterized by recurrent structural alterations, *EIF3E* dysregulation in human cancer appears to occur predominantly through altered expression and perturbation of selective translational programs.

A notable exception to this general pattern is represented by recurrent rearrangements involving members of the R-spondin family, which have emerged as an alternative mechanism of WNT pathway activation in human cancer. Among these, *EIF3E::RSPO2* is particularly intriguing from both biological and historical perspectives because it reconnects the human orthologues of int6 and int7, two loci originally identified independently as common MMTV integration sites [7,44,45], as illustrated in Figure 2.

The fusion was first identified in colorectal carcinoma through transcriptome sequencing of tumors lacking the canonical alterations that typically activate the WNT pathway [14]. Subsequent studies demonstrated that *EIF3E::RSPO2* represents a recurrent, although uncommon, molecular event present from the earliest stages of colorectal tumorigenesis, while larger series confirmed that RSPO2 fusions account for only a small fraction of colorectal carcinomas [45,46,47]. In a large study of 1019 colorectal cancers, RSPO fusions were identified in 29 cases (2.8%), whereas *EIF3E::RSPO2* was detected in only 5 cases, corresponding to approximately 0.5% of the entire cohort [45]. More recently, isolated cases have expanded the spectrum of associated neoplasms to include urachal adenocarcinoma and metastatic pancreatic ductal adenocarcinoma, indicating that this rearrangement is not restricted to colorectal tumorigenesis but may occur, albeit exceptionally, in other epithelial malignancies [48,49]. The recent description of an *EIF3E::RSPO2* fusion in a KRAS-wild-type metastatic pancreatic ductal adenocarcinoma further suggests that the rearrangement may identify a rare molecular subset driven by mechanisms distinct from the canonical genomic landscape of pancreatic cancer [49] (Table 1).

Molecular characterization of the fusion has provided important mechanistic insights. The canonical rearrangement results from an intrachromosomal deletion with breakpoints in intron 1 of *EIF3E* and intron 1 of *RSPO2*, thereby retaining the *EIF3E* promoter and exon 1 and juxtaposing them to *RSPO2* exon 2 and the downstream coding exons. The resulting chimeric transcript, rather than encoding a biologically relevant *EIF3E*–*RSPO2* fusion protein, drives ectopic expression of an intact and functional *RSPO2* protein under the transcriptional control of *EIF3E* regulatory elements [14,45].

This distinction is fundamental. In contrast to many oncogenic fusion genes in which the transforming activity resides within the fusion protein itself, the biological consequence of *EIF3E::RSPO2* appears to derive primarily from deregulated RSPO2 expression and subsequent potentiation of canonical WNT signalling. Experimental models have demonstrated that this mechanism is sufficient to initiate intestinal tumorigenesis and that the resulting tumors remain dependent on ligand-mediated WNT activation, providing a rationale for therapeutic targeting of the WNT pathway [50].

Although the molecular mechanism responsible for RSPO2 activation is now well established, the possible biological consequences of disrupting one EIF3E allele in fusion-positive tumors remain essentially unexplored. The canonical *EIF3E::RSPO2* fusion retains only the non-coding 5′ portion of EIF3E and does not generate a chimeric protein containing functional EIF3E domains [14]. Accordingly, no experimental evidence currently supports a direct contribution of EIF3E protein dysfunction to the oncogenic activity of the fusion. Nevertheless, several experimental studies indicate that EIF3E is dosage-sensitive. Heterozygous *Eif3e* knockout mice display reduced EIF3E expression associated with developmental and proliferative abnormalities, indicating that complete compensation by the remaining allele does not always occur under physiological conditions [51]. In addition, experimental reduction in EIF3E expression has been shown to affect multiple cellular processes involved in genome stability, epithelial homeostasis and tumor-related phenotypes [12,33,52]. Although these findings do not demonstrate that EIF3E haploinsufficiency contributes to the biology of *EIF3E::RSPO2*-positive tumors, they provide a biological rationale for investigating whether disruption of one EIF3E allele exerts subtle context-dependent effects. Clarifying whether altered EIF3E gene dosage influences tumor development or progression in fusion-positive cancers therefore represents an important question for future studies, as highlighted in Figure 3.

Available expression and functional studies do not currently provide direct evidence that disruption of one EIF3E allele results in functionally relevant EIF3E haploinsufficiency in fusion-positive tumors. Published patient series have consistently demonstrated increased RSPO2 expression associated with the *EIF3E::RSPO2* rearrangement, but they have not specifically assessed allele-specific EIF3E expression or demonstrated reduced EIF3E protein levels attributable to the rearranged allele. Similarly, experimental recreation of the intron 1–intron 1 deletion confirmed loss of the affected 3′*Eif3e* genomic region and robust activation of *Rspo2*, but it did not establish a contribution of reduced *Eif3e* gene dosage to the resulting tumor phenotype. Thus, although disruption of one EIF3E allele is an inherent structural consequence of the canonical rearrangement, its functional significance remains unproven. Dedicated analyses of EIF3E mRNA and protein expression, ideally incorporating allele-specific approaches in molecularly confirmed fusion-positive tumors and appropriate controls, will be required to determine whether biologically relevant EIF3E haploinsufficiency actually occurs. Validation in patient material is additionally complicated by the rarity of the fusion, the limited ability of bulk tumor assays to resolve allele-specific effects, intratumoral heterogeneity, and variable stromal admixture.

From a historical perspective, *EIF3E::RSPO2* represents a remarkable convergence of two independent lines of investigation. The original MMTV insertional mutagenesis studies identified int6 and int7 as distinct common integration sites associated with murine mammary tumorigenesis [7,44]. Their subsequent characterization independently linked these loci to EIF3E, a multifunctional regulator of selective translation and cellular homeostasis, and RSPO2, a potent enhancer of canonical WNT signaling. Their unexpected reunion in a recurrent human cancer rearrangement therefore establishes a direct historical connection between classical retroviral genetics and modern cancer genomics [14].

Importantly, this historical convergence should not be interpreted as evidence that EIF3E protein directly contributes to the transforming activity of the fusion. Current evidence indicates that the oncogenic mechanism is primarily mediated by transcriptional activation of RSPO2 through the 5′ regulatory region of EIF3E [14,45,50]. Nevertheless, the fusion provides an extraordinary conceptual bridge between classical retroviral insertional mutagenesis and modern cancer genomics, illustrating how discoveries originating from experimental mouse models continue to illuminate previously unrecognized mechanisms of human tumorigenesis.

## 3. Future Directions

Although genomic studies have profoundly advanced our understanding of cancer biology, genomic information alone cannot fully explain how cellular phenotypes emerge from genetic alterations. Increasing evidence indicates that the biological consequences of genomic events are critically shaped by additional regulatory layers, including epigenomic, epitranscriptomic and translational mechanisms [53,54,55]. The evolving biological profile of EIF3E places this molecule at a particularly interesting interface between these different levels of regulation and raises new questions that extend well beyond its genomic alterations in cancer.

A direct connection between epigenomic regulation and EIF3E has recently been demonstrated in t(4;14) multiple myeloma, where the lysine methyltransferase REIIBP remodels chromatin by altering H3K4me3 and H3K27me3 occupancy at the EIF3E locus, resulting in increased EIF3E expression. Rather than globally enhancing protein synthesis, elevated EIF3E selectively promotes translation of TLR7, activates the BTK–NF-κB signalling pathway and contributes to a pro-inflammatory tumour phenotype [56]. These findings identify EIF3E as a molecular effector through which pathological chromatin remodelling can be converted into selective translational outputs and clinically relevant cellular phenotypes.

Recent studies have further expanded this concept by demonstrating that EIF3E functions as a translational checkpoint required to maintain immune homeostasis. Genetic ablation of *Eif3e* in activated B lymphocytes disrupts selective translational programmes and increases lymphoma susceptibility [15]. Remarkably, tumour development results not from direct transformation of *Eif3e*-deficient cells but from pathological remodelling of the immune microenvironment that promotes malignant transformation of neighbouring lymphocytes. These observations extend the biological significance of EIF3E beyond cell-autonomous translational regulation to the maintenance of tissue homeostasis and intercellular communication.

More broadly, selective translation is increasingly recognized as a major downstream effector through which epigenomic and epitranscriptomic programmes are converted into functional cellular phenotypes. Interactions between components of the eIF3 complex and N6-methyladenosine (m6A)-modified transcripts, together with the functional cooperation between epitranscriptomic regulators and the translational machinery, provide a conceptual framework linking chromatin regulation, RNA modification and selective protein synthesis [57,58,59,60,61]. Although these mechanisms have not yet been specifically demonstrated for EIF3E, they define an important area for future investigation. In particular, determining whether EIF3E participates in the integration of epigenomic, epitranscriptomic and translational regulatory networks represents one of the most compelling questions emerging from current knowledge.

Several mechanistically distinct links between m6A-dependent RNA regulation and the eIF3 complex have been described. m6A residues within the 5′ untranslated region can directly recruit eIF3 and promote cap-independent translation, particularly under cellular stress conditions. This interaction appears to involve a multisubunit interface within eIF3 rather than a single dedicated subunit. In parallel, m6A reader proteins can facilitate translation through functional interactions with eIF3, while the m6A methyltransferase METTL3 has been reported to interact with eIF3h and enhance translation of selected transcripts. These observations establish multiple routes through which epitranscriptomic information can converge on the translation-initiation machinery. Importantly, however, the eIF3 subunits implicated in these mechanisms have predominantly included eIF3a, eIF3b, eIF3c, eIF3d, eIF3g, eIF3h and eIF3i, and a specific role for EIF3E in m6A recognition or m6A-dependent translation has not been demonstrated. Whether EIF3E influences these processes indirectly through its structural and regulatory functions within the eIF3 complex, or participates in distinct epitranscriptome-sensitive translational programs, therefore remains an open question.

This hypothesis could be directly tested by integrating m6A profiling with EIF3E-dependent translatome analyses, for example by comparing m6A-modified transcripts with changes in ribosome occupancy following EIF3E depletion or overexpression. EIF3E-associated RNA profiling and perturbation of m6A writers, erasers or readers could further determine whether specific epitranscriptomic marks influence the recruitment or translational efficiency of EIF3E-sensitive transcripts.

Collectively, these discoveries indicate that future investigations should move beyond the analysis of genomic alterations alone and adopt integrated approaches combining genomics, epigenomics, epitranscriptomics, transcriptomics, translatomics and proteomics. Such studies will be essential to define how EIF3E-dependent regulatory programmes are established in different cellular contexts, how they become dysregulated during tumorigenesis, and whether they can be therapeutically targeted through modulation of chromatin regulators, selective translation or downstream signalling pathways.

From a therapeutic perspective, the context-dependent functions of EIF3E argue against considering it, at present, as a conventional direct drug target. Rather, its role in selective translation may identify tumor-specific translational dependencies or downstream signaling pathways that could be therapeutically exploited. This distinction is particularly relevant for *EIF3E::RSPO2*-positive tumors, in which the established oncogenic event is RSPO2 overexpression and consequent WNT pathway activation, making the RSPO–WNT axis a more directly supported therapeutic vulnerability than EIF3E itself. More broadly, defining the EIF3E-dependent translatome in specific tumor contexts may reveal selectively translated oncogenic or stress-adaptation programs amenable to pharmacological intervention. Such approaches may ultimately provide greater specificity than global inhibition of translation initiation, which would be expected to affect essential protein synthesis in normal cells.

An additional priority will be to determine how EIF3E-dependent translational and proteostatic programs influence the tumor microenvironment. The emerging links between EIF3E, hypoxic and angiogenic responses, and immune homeostasis suggest that EIF3E perturbation may affect tumor development not only through cell-autonomous mechanisms but also by modifying vascular, inflammatory and immune interactions within the surrounding tissue.

Several limitations of the current evidence should nevertheless be acknowledged. First, the biological functions attributed to EIF3E have been derived from heterogeneous experimental systems, including yeast, cultured cells, genetically modified mice, and different human tumor types, which may partly account for the apparently divergent phenotypes reported across studies. Second, several mechanistic observations remain context-specific and have not yet been systematically validated across tumor types or in large patient cohorts. This is particularly relevant to the proposed consequences of EIF3E disruption in *EIF3E::RSPO2*-positive tumors, for which direct evidence of EIF3E haploinsufficiency is currently lacking. Similarly, the emerging connections between EIF3E, epigenomic regulation, and epitranscriptomic control remain incompletely defined, with several mechanisms established for the broader eIF3 complex but not specifically for EIF3E. Finally, the clinical and therapeutic implications of EIF3E-dependent selective translation remain largely exploratory. These limitations underscore the need for integrated functional studies and well-characterized patient cohorts to distinguish context-specific observations from conserved EIF3E-dependent mechanisms.

The history of EIF3E therefore mirrors the evolution of modern cancer biology. The first generation of studies identified cancer-associated genes; genomics subsequently revealed their structural alterations and molecular interactions. The next frontier is shifting toward understanding how genomic information is interpreted through interconnected layers of epigenomic, epitranscriptomic and translational regulation. Rather than representing the endpoint of a historical journey, the story of EIF3E illustrates how successive technological advances have revealed previously unrecognized dimensions of its biological function. There is every reason to believe that this scientific journey is still far from complete.

## 4. Conclusions

Over three decades of investigation have transformed the interpretation of EIF3E from that of a component of the general translation initiation machinery into that of a multifunctional regulator of selective translation, protein homeostasis and cellular physiology. The diverse consequences of EIF3E dysregulation, extending from altered proteostasis and genome stability to tissue and immune homeostasis, help explain its complex and context-dependent relationship with cancer.

The recent demonstration that *Eif3e* deficiency predisposes to lymphoid malignancy in vivo further establishes the physiological relevance of EIF3E-dependent translational regulation. In parallel, the identification of recurrent *EIF3E::RSPO2* fusions in human solid tumors has unexpectedly reunited int6 and int7, two genes originally discovered independently through MMTV insertional mutagenesis. While activation of RSPO2 represents the established oncogenic mechanism of this rearrangement, the possible consequences of disrupting one EIF3E allele remain unresolved.

Taken together, these findings illustrate how the biological significance of EIF3E has continued to expand with advances in experimental and genomic technologies, while emphasizing that important aspects of its role in cancer remain to be defined.

## Figures and Tables

**Figure 1:**
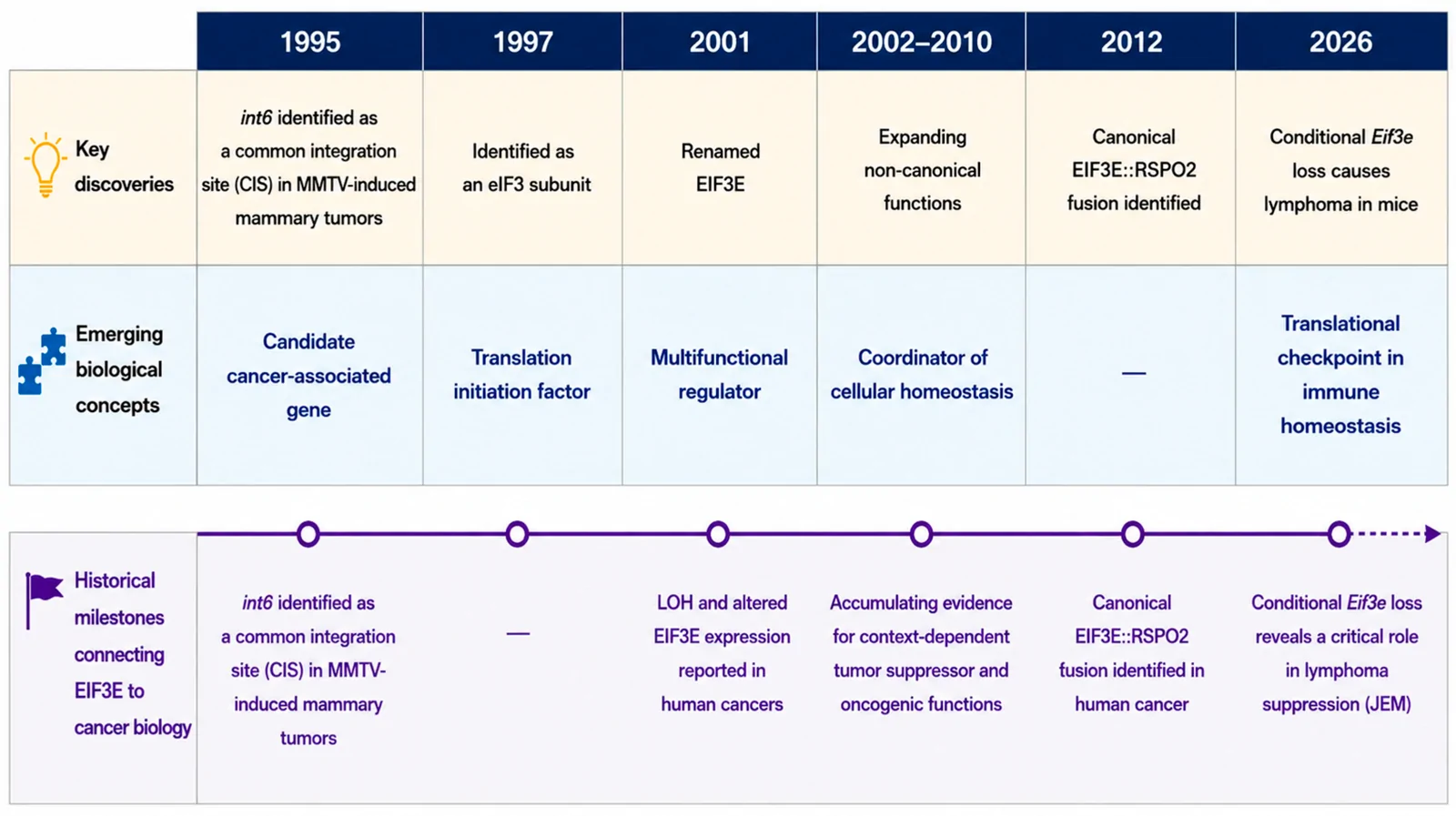
Chronological timeline of key discoveries and concepts in EIF3E biology and cancer. The figure summarizes major milestones from the identification of *int6* as a common integration site in MMTV-induced mammary tumors [7] to the recent demonstration that conditional *Eif3e* loss promotes lymphoma development in mice. The progressive characterization of EIF3E revealed multiple non-canonical functions, including proteostatic regulation of HIF-2α [11] and involvement in the DNA damage response, genome stability, and cellular senescence [12,13]. The identification of the canonical *EIF3E::RSPO2* fusion in 2012 [14] provided an unexpected link between the original retroviral insertional studies and human cancer genomics, while recent conditional *Eif3e* models established a role for EIF3E-dependent translational control in immune homeostasis and lymphoma suppression [15].

**Figure 2:**
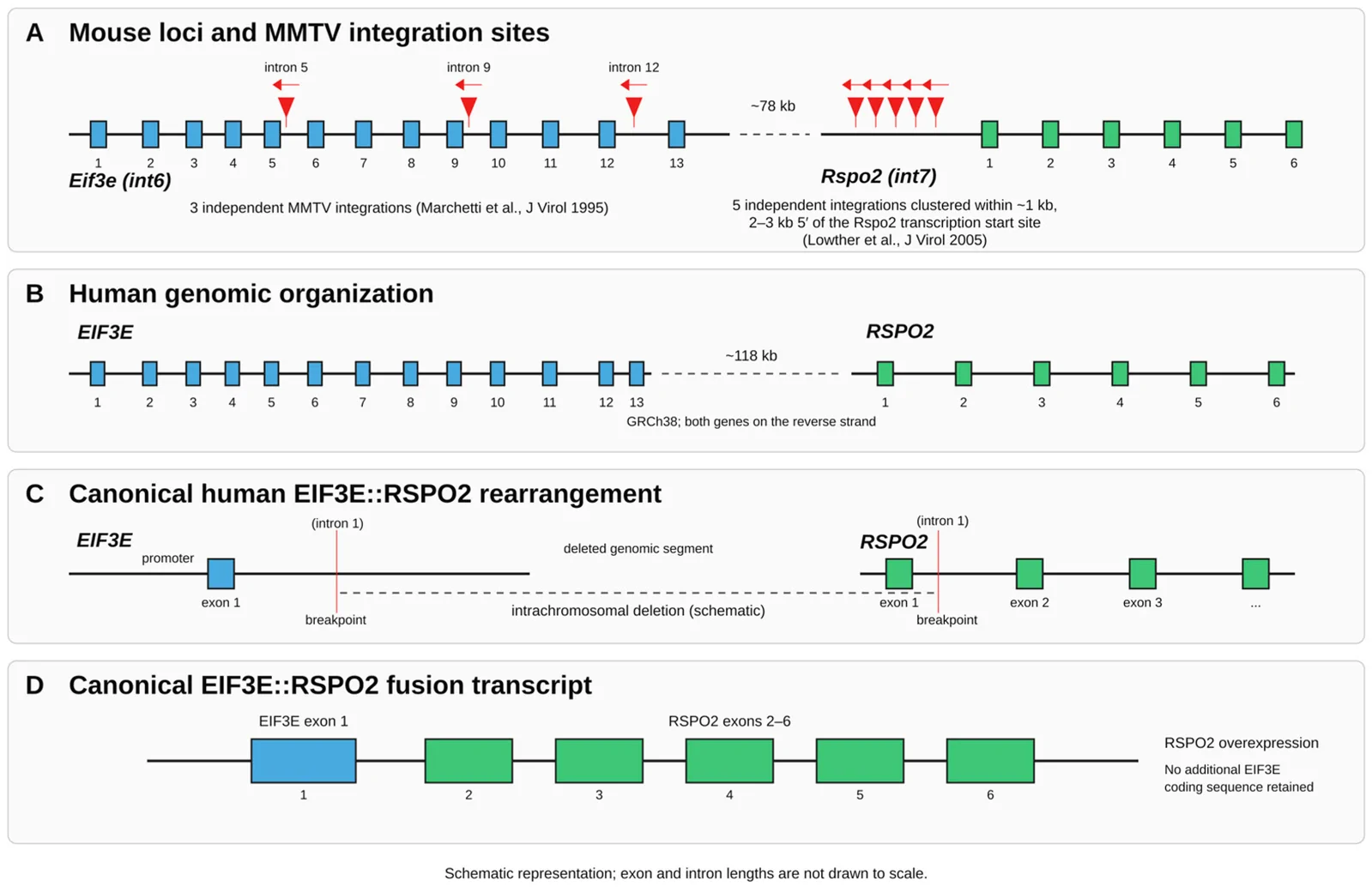
From the conserved int6–int7 genomic neighborhood to the canonical *EIF3E::RSPO2* fusion in human cancer. (**A**) In the mouse genome, *Eif3e* (int6) and *Rspo2* (int7) are located within the same conserved genomic region on chromosome 15 and are separated by approximately 78 kb. Three independent MMTV integrations were identified within introns 5, 9, and 12 of *Eif3e* [7], whereas five independent integrations associated with *Rspo2* were clustered within an approximately 1-kb region located 2–3 kb upstream of its transcription start site [44]. (**B**) In humans, *EIF3E* and *RSPO2* retain the same genomic neighborhood on chromosome 8q23.1 and are separated by approximately 118 kb. (**C**) The canonical *EIF3E::RSPO2* rearrangement results from an intrachromosomal deletion with breakpoints in intron 1 of *EIF3E* and intron 1 of *RSPO2*. The rearrangement retains the EIF3E promoter and exon 1 while deleting the intervening genomic region and *RSPO2* exon 1, thereby placing *RSPO2* exon 2 and the downstream coding exons under EIF3E transcriptional control. (**D**) The resulting chimeric transcript joins EIF3E exon 1 to RSPO2 exon 2 and drives aberrant expression of an intact RSPO2 coding sequence, without generating a biologically relevant EIF3E–RSPO2 fusion protein. Reference transcripts used for the schematic representation are mouse *Eif3e* NM_008388.2 and *Rspo2* NM_001357957.1, and human *EIF3E* NM_001568.3 and *RSPO2* NM_178565.5. Schematic representation; exon and intron lengths and relative genomic distances are not drawn to scale.

**Figure 3:**
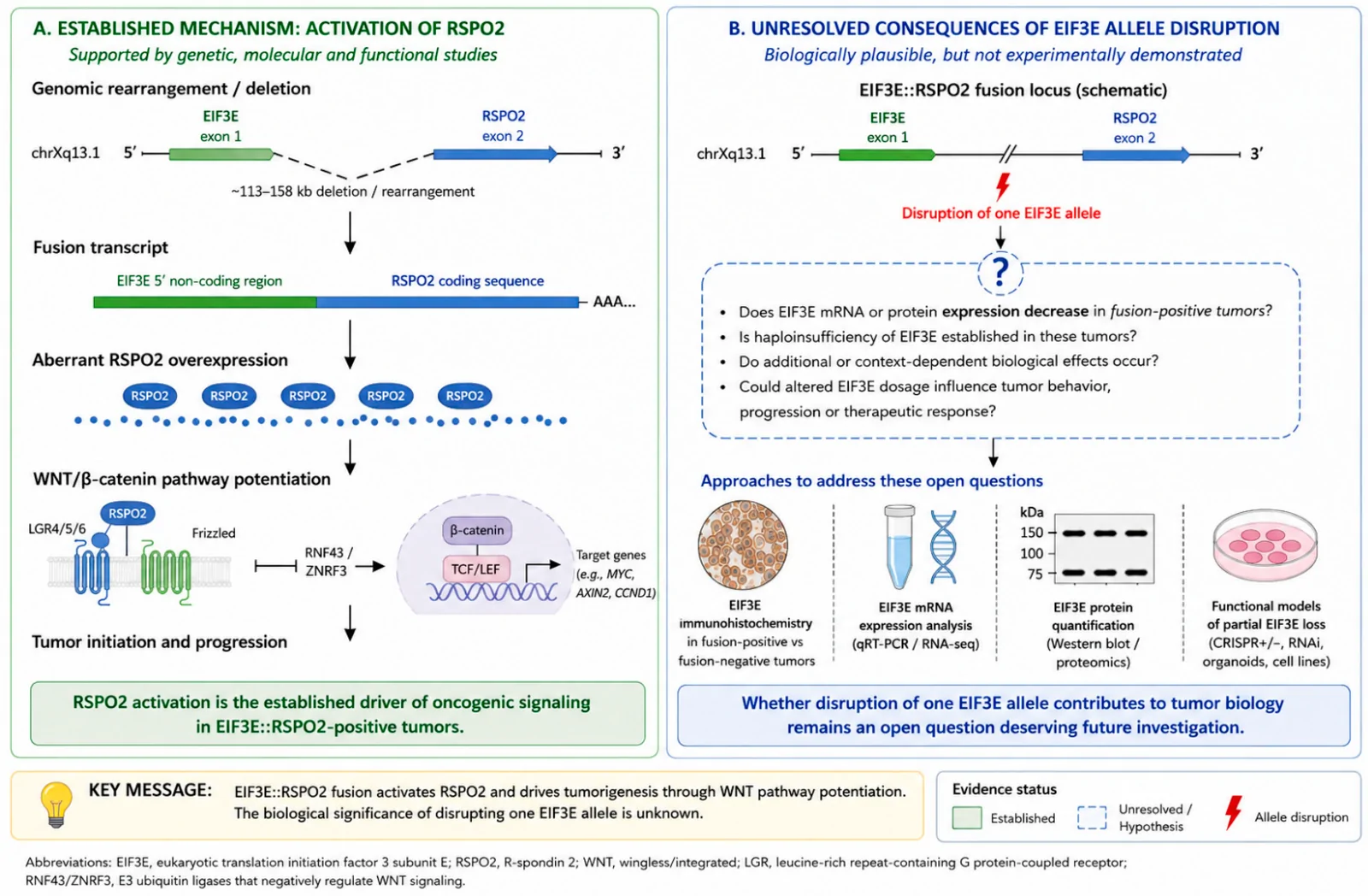
Established mechanism of RSPO2 activation and unresolved biological consequences of EIF3E disruption in canonical *EIF3E::RSPO2* fusions. (**A**) The canonical *EIF3E::RSPO2* rearrangement activates RSPO2 by placing its coding sequence under the control of the constitutively active EIF3E promoter. This promoter hijacking mechanism results in aberrant RSPO2 overexpression, potentiation of WNT/β-catenin signaling, and tumorigenesis, and is now considered the established oncogenic mechanism of the fusion. (**B**) In contrast, the biological significance of disruption of one EIF3E allele remains unknown. Because the canonical rearrangement retains only the non-coding 5′ portion of EIF3E and does not generate a chimeric protein containing functional EIF3E domains, no experimental evidence currently supports a direct contribution of EIF3E protein dysfunction to tumor development. Based on the known dosage sensitivity of EIF3E, haploinsufficiency represents a plausible but unproven hypothesis. The figure summarizes the principal experimental approaches—including immunohistochemical, transcriptomic, protein expression, and functional studies—that may clarify whether EIF3E disruption contributes to tumor biology beyond RSPO2 activation.

**Table 1:** Published reports of *EIF3E::RSPO2* fusion in human tumors and their conceptual contribution.

Reference	Tumor Type	Main Finding	Conceptual Contribution
Seshagiri et al. Nature. 2012 [14]	Colorectal carcinoma	First identification of recurrent *EIF3E::RSPO2* fusion and RSPO2 activation by promoter hijacking.	Established *EIF3E::RSPO2* as a novel WNT-activating mechanism in colorectal cancer.
Hashimoto et al. Histopathology. 2019 [46]	Traditional serrated adenoma	Identification of *EIF3E::RSPO2* in precursor serrated lesions.	Suggested that the fusion may occur early during serrated colorectal tumorigenesis.
Jha et al. Genes Chromosomes Cancer. 2024 [48]	Urachal adenocarcinoma	First non-colorectal epithelial tumor harboring *EIF3E::RSPO2*.	Expanded the histological spectrum beyond colorectal neoplasia.
Sayagués JM et al. Int J Mol Sci. 2026 [49]	Metastatic pancreatic ductal adenocarcinoma (KRAS wild type) (single case report)	First *EIF3E::RSPO2*-positive pancreatic ductal adenocarcinoma.	Identified *EIF3E::RSPO2* as a potential rare KRAS-independent molecular subset and therapeutic candidate.

Abbreviations: KRAS, Kirsten rat sarcoma viral oncogene homolog; RSPO2, Roof plate-Specific Sponding-2.

## Data Availability

No new data were created or analyzed in this study. Data sharing is not applicable to this article.
